# Remote Induction Heating of Metal Implants for Noninvasive Elimination of *Pseudomonas aeruginosa* Biofilm, Augmented by Tobramycin

**DOI:** 10.3390/microorganisms14102277

**Published:** 2026-10-06

**Authors:** Haydar A. S. Aljaafari, Parham Parnian, Nadia I. Abdulwahhab, Jaymes Van Dyne, Eric Nuxoll

**Affiliations:** 1 Department of Chemical and Biochemical Engineering, University of Iowa, Iowa City, IA 52242, USA; haydar-aljaafari@uiowa.edu (H.A.S.A.); parham-parnian@uiowa.edu (P.P.); nadia.i.abdulwahhab@uotechnology.edu.iq (N.I.A.); jaymes-vandyne@uiowa.edu (J.V.D.); 2 College of Chemical Engineering, University of Technology, Baghdad P.O. Box 18310, Iraq; 3 College of Applied Sciences, University of Technology, Baghdad P.O. Box 18310, Iraq

**Keywords:** medical implant infection, induction heating, hyperthermia, thermal shock, prosthesis-related infections

## Abstract

Thermal shock is a promising new approach to eliminating biofilm infections on medical implants without surgically replacing the device. Most studies determining the minimum necessary thermal shock used immersion in hot media to provide precise, rapid, uniform temperature control. In vivo, however, this shock must be focused directly on the implant surface, raising the concern that bacteria may partially evade the shock by momentarily dispersing into the cooler surroundings. This study tests that hypothesis using remote induction heating, in which *Pseudomonas aeruginosa* biofilms were thermally shocked by an external alternating magnetic field heating the substrate directly. It also assessed the impact of tobramycin on the minimum thermal shock required. Susceptibility to tobramycin alone, thermal shock alone, and their combination was characterized over temperatures ranging from 50 to 80 °C and duration of 1 to 30 min with biofilms of two distinctly different architectures and population densities. Remote heating was then evaluated on biofilms in a flow cell where media was continuously replaced to keep its temperature below the biofilm’s. Comparison with immersion heating showed that, despite losing 90% of the biofilm to dispersion into the flushed media, biofilms thermally shocked in the flow cell had significantly higher populations than biofilms comparably shocked by immersion, though biofilms could still be completely eliminated via remote heating. Tobramycin alone did not eliminate biofilm even at clinically infeasible concentrations but, when combined with thermal shock, achieved complete elimination at milder thermal shocks, indicating a complex synergy also observed with antibiotics of another class. Remote heating successfully eliminated biofilms while delivering heat in a clinically implementable manner. As an in vitro, these findings did not directly assess thermal damage to surrounding tissue, which requires further investigation in future treatment design.

## 1. Introduction

Infection of medical implants is one of the most challenging complications in current healthcare, affecting a growing number of patients and straining healthcare systems. These infections occur when bacteria colonize the implant surface, forming a biofilm that confers mechanical stability, antimicrobial tolerance, and protection from host immune response. *Pseudomonas aeruginosa* is well known for its association with pathogen biofilm formation on implants, due to its resistance mechanisms, metabolic versatility, and capacity to colonize abiotic surfaces [1,2]. Bacteria within a biofilm tolerate much higher antibiotic concentrations than those required for eliminating their planktonic counterparts. This tolerance results from the restriction of antibiotic penetration through the matrix, metabolically dormant persister cells, and stress responses within the bacterial community [3,4,5]. Consequently, biofilm infections on medical implants are difficult to resolve with antibiotics alone, and the current standard treatment remains surgical removal and replacement of the implant, followed by long-term antibiotic therapy [6,7,8]. This approach is associated with an increased rate of morbidity, extended hospitalization, and elevated risk of reinfection of the replacement implant [9,10,11,12,13]. Despite decades of research aimed at preventing biofilm formation or eradicating established biofilms [14,15,16,17,18,19], no approach has solved the biofilm infection problem in clinical settings, highlighting the need for new mitigation strategies.

Thermal shock has recently emerged as one such strategy, showing promising results in eliminating biofilm infections [20,21,22,23]. The severity of the thermal shock must be balanced between successfully eliminating the biofilm and minimizing the damage to surrounding tissue, hence the importance of characterizing the biofilm’s thermal susceptibility. Previous studies characterized thermal susceptibility of *P. aeruginosa* biofilms by immersion in heated media, quantifying temperature and time-dependent reduction in population density up to complete elimination [24,25]. Reincubation studies of thermally shocked biofilms indicated that reductions by over four orders of magnitude rendered the biofilm unviable regardless of the temperature–time combination achieving the reduction [21,25,26]. In addition, the combination between thermal shock and antibiotics was investigated. Although antibiotics alone cannot eliminate the biofilm, their contribution to the overall population reduction can help achieve complete elimination at milder thermal shocks [21,27]. In *P. aeruginosa*, their combined effect appeared additive [26], indicating orthogonal mechanisms. In contrast, population reductions from combined treatment in *Staphylococcus epidermidis* exceeded the sum of the individual treatments, indicating synergistic mechanisms that depend on the organism [20].

Infected implants cannot be immersed in heated media in situ, so delivering the thermal shock precisely to their point of application while minimizing the exposure of the surrounding tissue is critical [28]. For implants with wireless telemetry and onboard power supply, this heat can be generated electroresistively at the implant surface or pumped thermoelectrically from deeper in the implant [29], creating a cool sink for the heat to return to after the shock. Wireless induction heating eliminates the need for an onboard power supply. As long as the surface upon which the biofilm is growing is electrically conductive (i.e., metal) [23] or contains magnetically susceptible material (e.g., superparamagnetic magnetite nanoparticles) [30], it will heat up in the presence of an alternating magnetic field. While induction heating has been demonstrated to reduce biofilm on metal for *S. epidermidis*, *E. coli*, *P. aeruginosa*, and *S. aureus* [23,31,32,33,34], these studies were performed under static conditions in which the medium surrounding the implant was similarly heated by conduction from the heated substrate. In practice, it is hoped that the surrounding tissue will be kept at a lower temperature by convecting heat away from the implant surface via the circulatory system. Recent studies, however, indicate that at any given moment, a significant fraction of the bacteria are dispersed in the biofilm’s surroundings, rapidly interchanging with the biofilm itself [29,35]. The bacteria, then, may experience less cumulative thermal shock than delivered in previous studies, but may also be similarly convected away. The present work investigates this difference by using a flow cell to provide the convective transport. Tobramycin is also used to investigate the combined effect of thermal shock with antibiotics in this scenario. Tobramycin, an aminoglycoside antibiotic that inhibits ribosomal functionality, is clinically important for the treatment of *P. aeruginosa* infections, representing a frontline agent for respiratory and implant infections caused by this pathogen [36,37]. Prior studies have also demonstrated that a biofilm’s thermal susceptibility is strongly impacted by its culture conditions and resulting population density and architecture [25]. As this can vary clinically, two opposing extremes of biofilm culture were used. Drip flow reactor (DFR) biofilms allow a slow drip of fresh media to flow along the biofilm at a 10° incline, allowing rapid gas transport, continuous nutrient supply, and continuous waste removal, producing thick (>100 μm) carpet-like biofilms with a population density around 10^8.5^ CFU/cm^2^. Shaker table (ST) biofilms, by contrast, are grown in a finite volume of media with no waste removal, producing population densities closer to 10^7^ CFU/cm^2^.

In this study, we first characterize the susceptibility of *P. aeruginosa* biofilms cultivated using shaker table and drip flow reactor protocols to tobramycin alone, to thermal shock by immersion, and to the combination of tobramycin with thermal shock. We then evaluate remote induction heating of biofilm on stainless steel coupons within a flow cell in which a continuously circulating medium maintains a lower temperature than that of the metal surface and compare it directly against immersion heating. Together, these findings establish remote induction heating as a promising, noninvasive, and clinically applicable approach for treating biofilm infections on metal implants without the need for surgical intervention.

## 2. Materials and Methods

### 2.1. Streak and Inoculum

*P. aeruginosa PAO1* (ATCC 15692, American Type Culture Collection, Manassas, VA, USA) was streaked onto a nutrient agar (Difco, Sparks, MD, USA) and grown for 24 h at 37 °C with plate inverted. Using a sterile loop, two isolated colonies were transferred into 5 mL tryptic soy broth (30 g/L; TSB, Becton, Dickinson and Company, Franklin Lakes, NJ, USA) and cultured for 24 h at 37 °C to produce an inoculum of approximately 10^9^ CFU/mL.

### 2.2. Biofilm Growth Protocols

Two protocols were used to produce biofilms of distinct population density and architecture, consistent with prior studies [26].

#### 2.2.1. Shaker Table (ST) Biofilm

For immersion experiments, biofilms were grown on fully frosted glass microscope slides (75 × 25 × 1 mm, Leica Biosystems, Buffalo Grove, IL, USA). Each slide was placed in a well of a four-well dish, with each well holding 5 mL of TSB (30 g/L) and 0.333 mL of inoculum, sealed with parafilm, and incubated at 37 °C for 96 h under orbital shaking (VWR 1000, 15 mm orbit) at 160 rpm.

For remote induction heating experiments, biofilms were cultured on custom-cut medical-grade 316L stainless steel coupons measuring 1 × 1 × 0.075 inch (25.4 × 25.4 × 1.91 mm) (McMasterCarr, Elmhurst, IL, USA) using the same protocol.

#### 2.2.2. Drip Flow Reactor (DFR) Biofilm

DFR biofilms were prepared on frosted glass slides, which were placed in a four-well drip flow reactor (Biosurface Technologies, Bozeman, MT, USA) with 15 mL of 30 g/L TSB and 1 mL of inoculum per well. The reactor was first run horizontally at 37 °C for 4 h batch phase, it was then inclined 10°, and a continuous trickle of 3 g/L TSB was applied at 1.25 L/day per well for 20 h at 37 °C.

### 2.3. Tobramycin Treatment

Tobramycin sulfate (Sigma-Aldrich, St. Louis, MO, USA) was dissolved in deionized water to 5 mg/mL, filter sterilized (0.22 µm PES membrane, Millex-GP), kept at 2 °C for up to 2 weeks, and diluted freshly for each experiment. Mature biofilms were rinsed for 1 min in 5 mL TSB (3 g/L) to remove nonattached bacteria, and then they were moved into fresh wells containing 5 mL TSB (30 g/L) and the target concentration of tobramycin. DFR biofilms were treated with 0.25 and 4 µg/mL. ST biofilms were treated with 0.25, 1, 4, 16, 64, or 256 µg/mL, investigating concentrations far beyond human systemic toxicity. A control biofilm was exposed to only tobramycin in each trial to confirm antibiotic activity.

### 2.4. Thermal Shock Alone by Water-Bath Immersion

Immersion provides almost instantaneous, uniform, precisely controlled thermal shock, providing a baseline for the effect of temperature on biofilms. Mature biofilms on glass slides were rinsed for 1 min in 5 mL TSB (3 g/L) before transferring to preheated four-well dishes containing 5 mL TSB (3 g/L) per well, held in a temperature-controlled water bath set to the target temperature throughout the shock. Shocks were applied at 50, 60, 70, or 80 °C for 1, 2, 5, 10, or 30 min and then immediately transferred into 5 mL of 3 g/L TSB at ambient temperature for resuspension and enumeration.

### 2.5. Combined Tobramycin and Immersion Thermal Shock

This protocol extends the immersion thermal shock of Section 2.4 by adding a tobramycin pre-, during, and post-thermal shock. Following a literature procedure established for ciprofloxacin [26], biofilms were first exposed to tobramycin (0.25 or 4 µg/mL) for 4 h at 37 °C and then transferred into 5 mL preheated TSB (3 g/L) containing an identical tobramycin concentration at the designated temperature for the designated shock duration. Once treated, biofilms were either immediately resuspended and enumerated or reincubated at 37 °C in fresh TSB at the corresponding tobramycin concentration to complete a 24 h total exposure. ST biofilms were shocked at 50 °C (5, 30 min), 60 °C (1, 5 min), or 70 °C (1 min). Due to their larger initial population density, DFR biofilms were shocked aggressively (60 °C for 5 or 10 min, 70 °C for 1 or 2 min). At least three biofilms were shocked under each condition.

### 2.6. Remote Induction Heating in a Flow Cell

**Figure 1:**
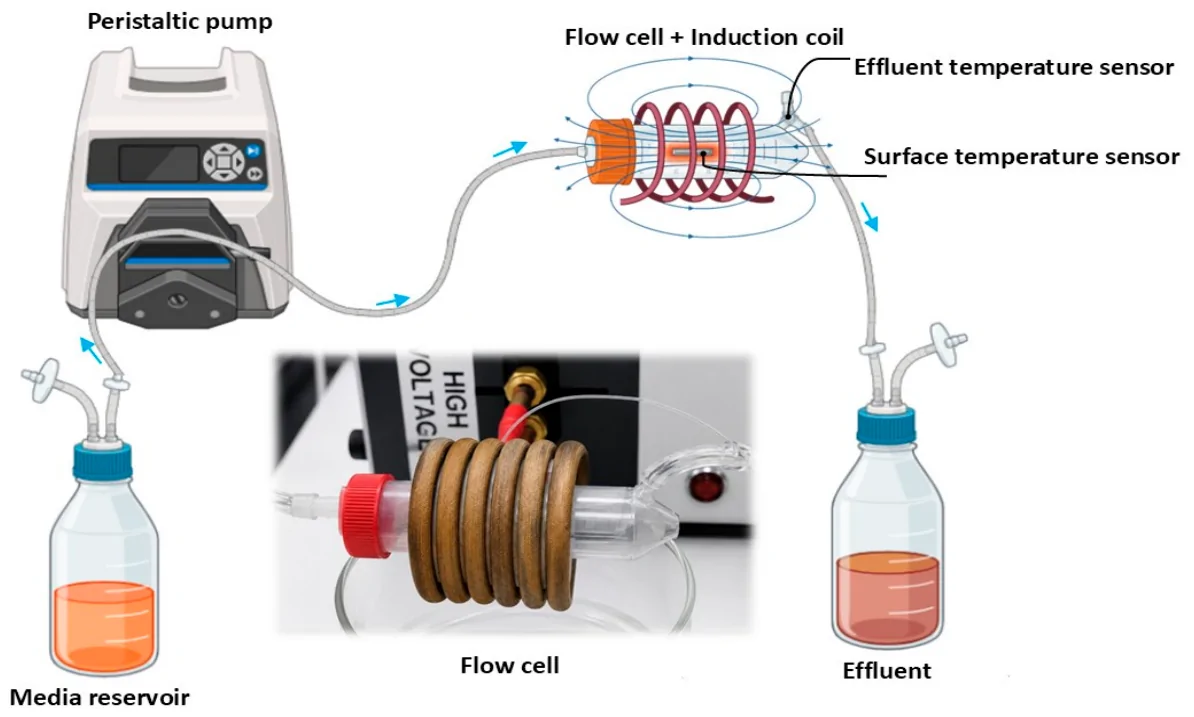
Schematic of the flow cell under an alternating magnetic field. Blue arrows indicate the direction of media flow through the system.

As induction heating requires electrically conductive or magnetically susceptible substrate, a steel coupon colonized by *P. aeruginosa* biofilm was placed within an alternating magnetic field (AMF), and the coupon was heated remotely by eddy currents induced within the metal itself. A custom-made AMF generator was used with an induction coil of 2.32 kA/m maximum field strength. The flow cell was constructed from a 50 mL conical centrifuge (Falcon, Thermo Fisher Scientific, Waltham, MA, USA) tube, which was positioned horizontally at the center of the AMF coil. To reduce dead volume, one third of the tube was filled with a two-component epoxy cured 24 h before first use. The cured epoxy occupied the lower portion of the cylindrical body in addition to the conical tip, defining the flow channel. This ensured that the medium flowed smoothly across the biofilm without stagnant pockets. Influent entered through a port in the cap, and effluent exited at the upper end of the opposite side of the tube. Dilute TSB (3 g/L) at room temperature was circulated at 100 mL/min using a peristaltic pump (L100-1S-2/YZ1515X, Longer Precision Pump, HE, Baoding, China). This flowing media served as a heat sink, limiting the temperature rise of the medium surrounding the heated coupon (see Figure 1). The coupon was positioned at the exact center of the tube, and its surface temperature and the effluent temperature were monitored with K-type thermocouples (5SRTC-TT-K-40-36, Omega Engineering, Norwalk, CT, USA) connected to a data acquisition device (USB-1608G-Series, Measurement Computing Corporation MCC, Norton, MA, USA) to be monitored with LabVIEW 13.0.1. AMF power was regulated in real time by a PID control algorithm implemented in LabVIEW 13.0.1 (National Instruments, Austin, TX, USA) to minimize ramp time and overshoot and to hold the surface temperature. Coupons were heat-controlled at 60, 70, or 80 °C for 1, 5, or 30 min, with 37 °C controls.

### 2.7. Enumeration

Biofilm cell density was quantified by resuspension and plating. The biofilms were moved into 5 mL TSB (3 g/L) and disaggregated by mechanical scraping and sonication for 10 min at 45 kHz (VWR Symphony, 9.5 L, Radnor, PA, USA). Suspensions were serially diluted in 10-fold and spot-plated (10 µL) onto nutrient agar. After 20 h at 37 °C, colonies were enumerated and expressed as log (CFU/unit area).

### 2.8. Statistical Analysis

Data are presented as mean ± standard deviation. Statistical comparisons between immersion heating and remote induction heating were performed for each thermal shock condition using two-tailed unpaired Welch’s *t* tests. Differences were considered statistically significant when *p* < 0.05.

## 3. Results

### 3.1. Tobramycin Exposure Without Thermal Shock

**Figure 2:**
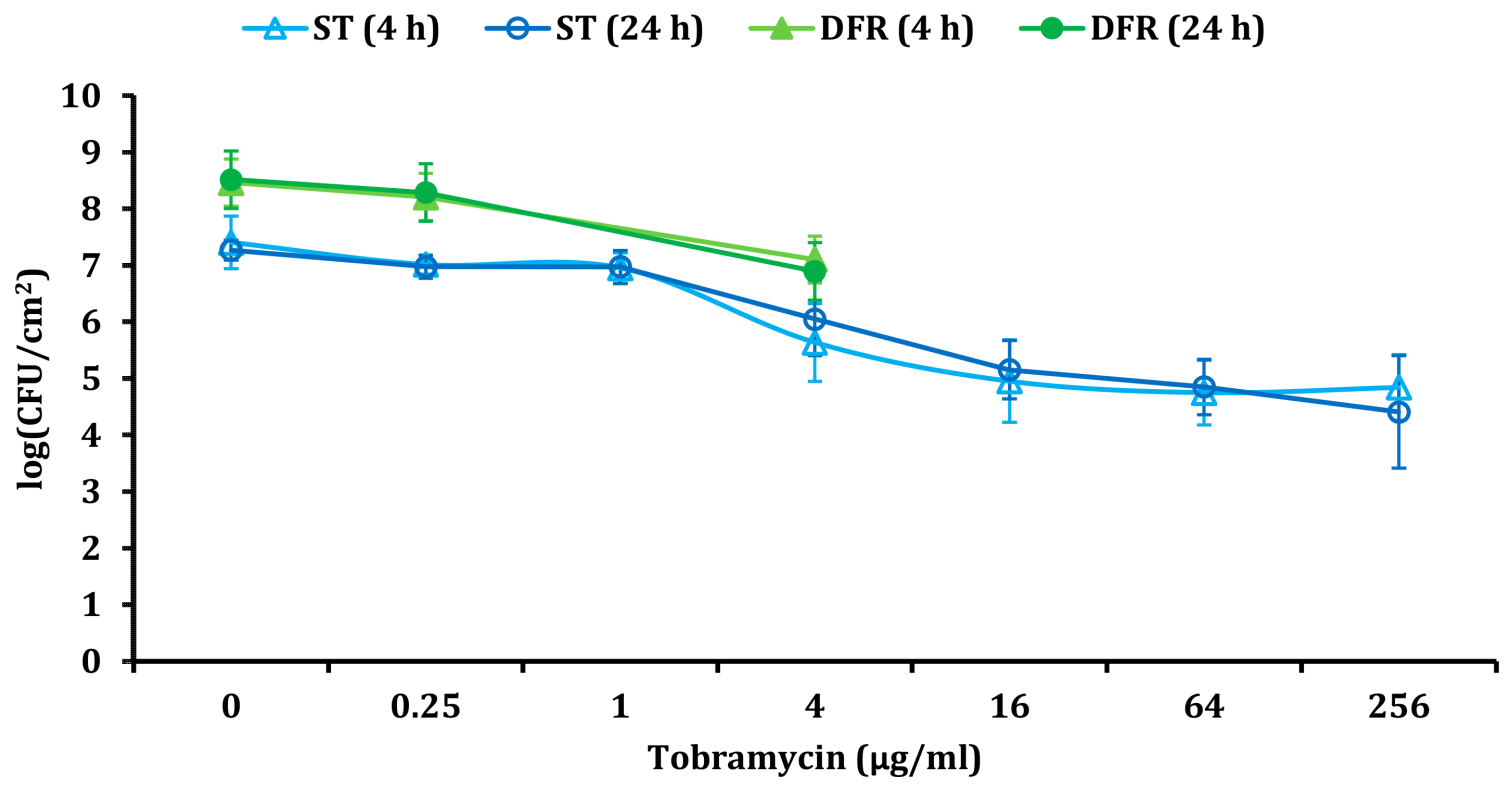
Tobramycin dose-response of (ST) and (DFR) *Pseudomonas aeruginosa* biofilm. Error bars indicate standard deviation across at least four biological replicates.

Tobramycin alone failed to eliminate *P. aeruginosa* biofilms of either architecture, even at concentrations far exceeding the physiological intravenous dose of 4 µg/mL. For ST biofilms, increasing tobramycin concentration from 0.25 to 256 µg/mL resulted in reducing the population density from 7.4 to 4.8 log (CFU/cm^2^) after 4 h of exposure, reaching 4.4 log(CFU/cm^2^) after an additional 20 h with little indication of approaching elimination. DFR biofilms were initially an order of magnitude more populous than ST biofilms and remained so with comparable antibiotic exposures, as seen in Figure 2.

### 3.2. Effects of Combined Tobramycin and Immersion Thermal Shock on Biofilm Viability

**Figure 3:**
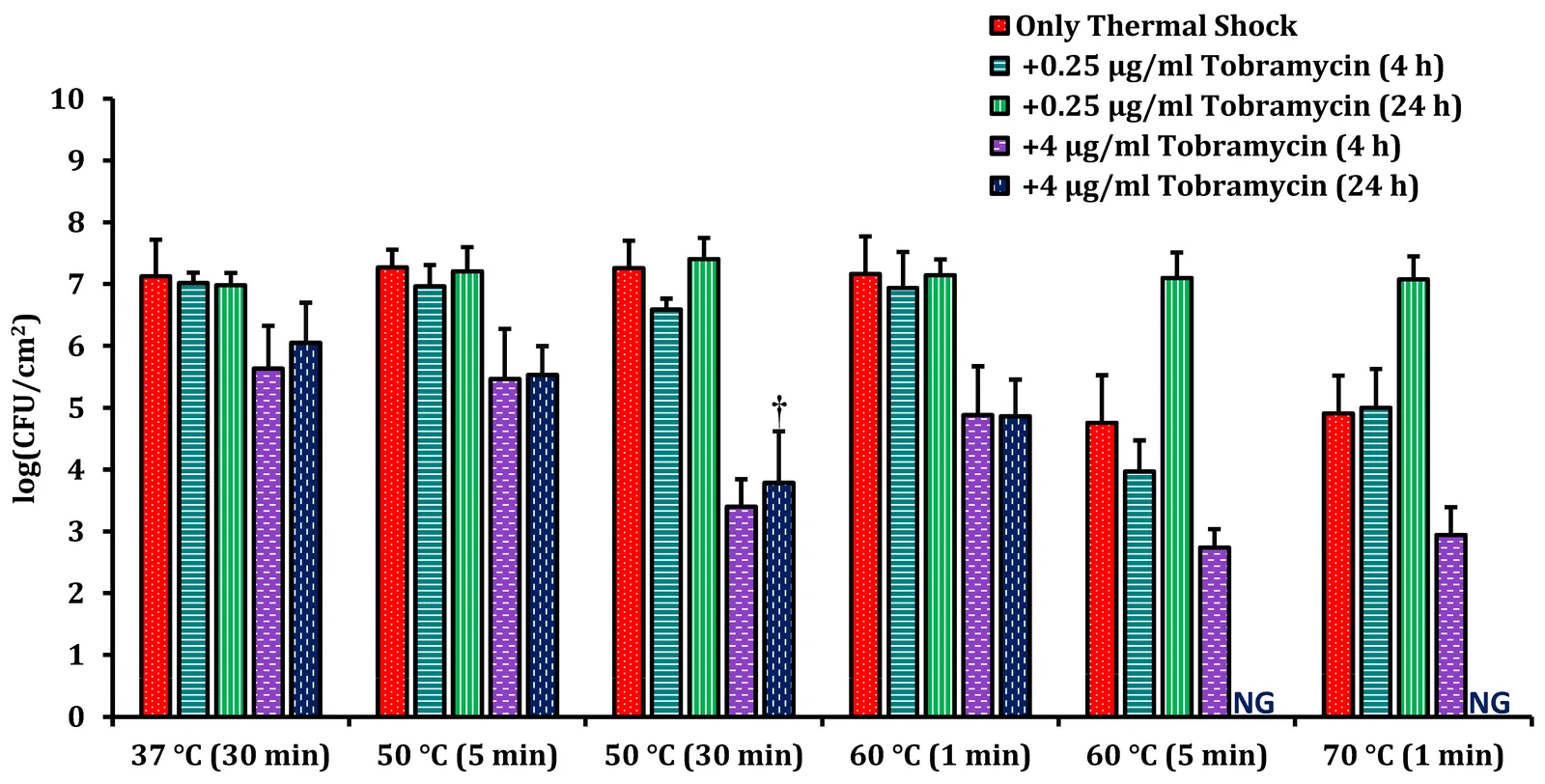
Combined tobramycin and thermal shock against ST *Pseudomonas aeruginosa* biofilm. Trials with no detectable colonies are indicated by “NG.” † Growth detected in only 17 of 24 samples. Error bars indicate standard deviation among at least six biological replicates.

For ST biofilms, thermal shock alone at 50 °C (5 or 30 min), 60 °C (1 or 5 min), and 70 °C (1 min) produced only partial reductions, and tobramycin alone at 0.25 or 4 µg/mL for both 4 and 24 h left the population above 5.5 log (CFU/cm^2^). In combination, the most aggressive pairings reached no detectable growth (NG), were 60 °C (5 min) with 4 µg/mL tobramycin and 24 h exposure, and 70 °C (1 min) with 4 µg/mL and 24 h. The 50 °C (30 min) combination with 4 µg/mL approached complete elimination, but with only 7 out of 24 tested samples. In each eliminated case, complete clearance required both the higher antibiotic concentration and the full 24 h reincubation (see Figure 3 and Table A1).

**Figure 4:**
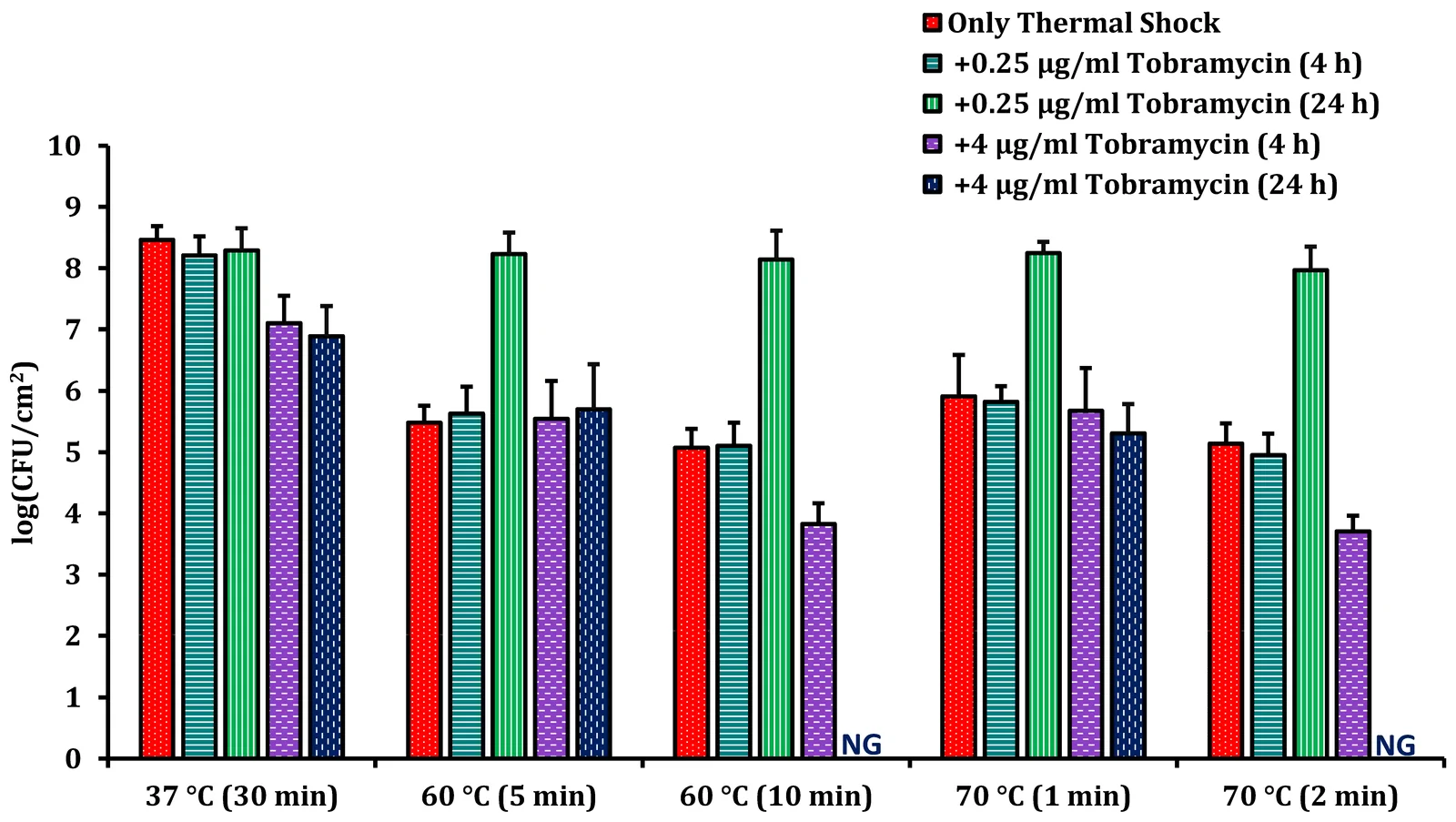
Combined tobramycin and thermal shock against DFR *Pseudomonas aeruginosa* biofilm. Trials with no detectable colonies are indicated by “NG.” Error bars indicate standard deviation among at least six biological replicates.

DFR biofilms, despite their higher initial population, were likewise eliminated by the most aggressive combinations. Of the conditions tested at 60 °C (5 or 10 min) and 70 °C (1 or 2 min), 60 °C (10 min) with 4 µg/mL tobramycin (24 h) and 70 °C (2 min) with 4 µg/mL tobramycin (24 h) reached NG, whereas milder shocks and shorter exposures left a residual population above 5.1 log (CFU/cm^2^). As with the ST biofilms, 4 μg/mL tobramycin enhanced thermal shock at each temperature, but biofilm elimination was achieved only with extended reincubation (see Figure 4 and Table A2).

### 3.3. Surface and Effluent Temperature During Remote Induction Heating

**Figure 5:**
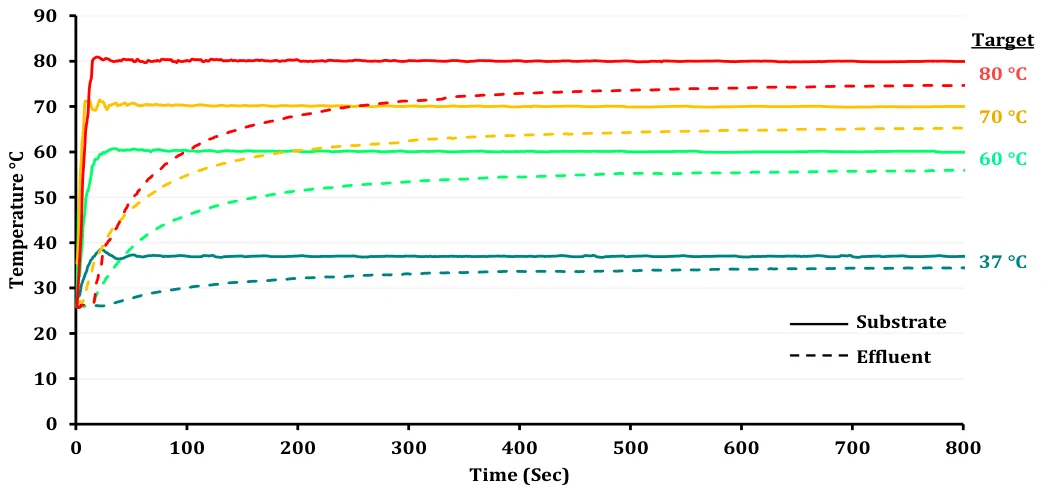
Surface and effluent temperatures during remote induction heating of the flow cell. Solid lines indicate substrate temperature; dashed lines indicate media temperature. Three replicates all gave essentially identical lines.

Remote induction heating selectively heated the coupon surface while the surrounding media remained substantially cooler. At each setpoint, the coupon surface reached its target temperature (60, 70, or 80 °C) within approximately 15 s and maintained it to within 1.5 °C, while the effluent media remained significantly cooler. Figure 5 shows the media still 9 °C cooler than the 80 °C substrate 5 min into the thermal shock, asymptotically approaching a 5 °C differential. Similar differentials are seen for the 70 °C substrate, shrinking slightly (4 °C) for the lowest temperature target (60 °C). These results indicate the minimum differential at the downstream exit; the media contacting the heated substrate at the upstream edge of the biofilm is much cooler (ambient). While the substrate was designed with large internal thermal transport to minimize the temperature gradient in the flow direction, any decrease in temperature at the upstream edge will decrease the observed efficacy of the treatment, making these results conservative vs. a substrate with a perfectly uniform temperature.

### 3.4. Efficacy of Remote Induction Heating and Comparison to Immersion

**Figure 6:**
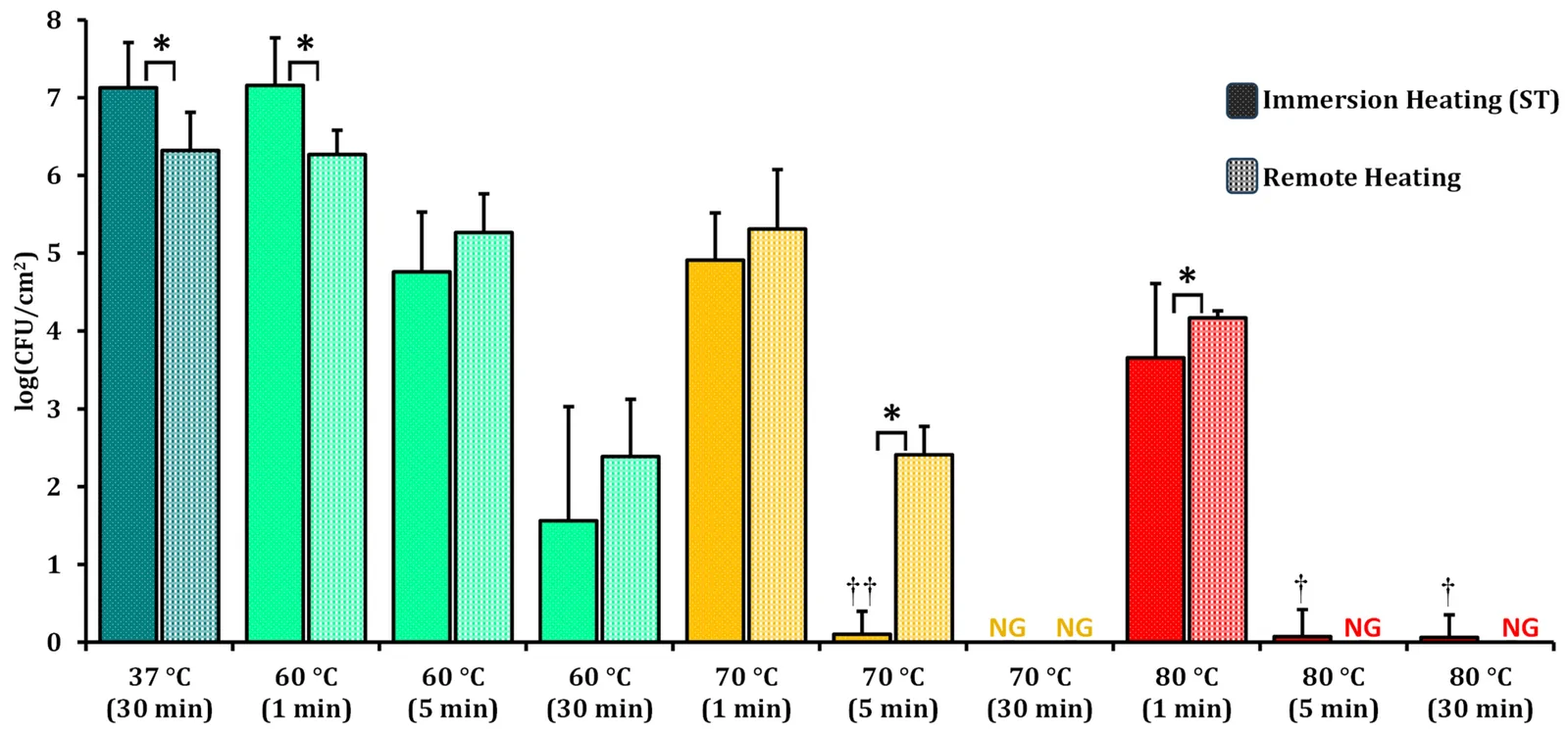
Comparison of remote induction heating versus water bath immersion against (ST) *Pseudomonas aeruginosa* biofilm. Trials with no detectable colonies are indicated by “NG.” † Growth detected in only 2 of 12 samples. †† Growth detected in only 1 of 8 samples. Error bars indicate standard deviation among at least three biological replicates. Asterisk indicates statistically significant differences between remote induction heating and immersion heating at the same thermal shock condition, as determined by two-tailed unpaired Welch’s *t* tests (*p* < 0.05).

The most aggressive temperature/exposure time combinations (70 °C for 30 min, 80 °C for 5 or 30 min) left no detectable CFU in any samples, regardless of which heating protocol was used (Figure 6). Slightly less aggressive shocks (60 °C for 30 min, 70 °C for 5 min, or 80 °C for 1 min) dropped the population density by several orders of magnitude using either heating protocol, but without reliably eliminating all CFU. Differences between the heating protocols are also apparent with these shocks, with the induction protocol leaving more detectable CFU than the immersion protocol, though the 60 °C 30 min results are too noisy to achieve significance. Milder shocks (60 °C for 5 min, 70 °C for 1 min) only reduce the population density by a couple orders of magnitude, and while the immersion protocol may appear to leave fewer CFU, the difference between protocols is not significant for these shocks. Importantly, though, at the mildest shocks (60 °C for 1 min and 37 °C for 30 min (i.e., control at incubation temperature)), the biofilms subjected to the induction heating protocol (even without temperatures above incubation) have significantly fewer CFU than the ones subjected to immersion heating. The order-of-magnitude reductions seen here must be due to induction-heating protocol steps other than actual heating.

## 4. Discussion

Biofilm infections on medical implants remain a major clinical problem, as conventional antibiotic therapy cannot eradicate the established biofilm. The current standard treatment involves surgical removal and replacement of the implant, which carries substantial morbidity, high cost, and an increased risk of reinfection. The present study investigates a noninvasive approach utilizing an alternating magnetic field, which can heat a metal or magnetically susceptible implant surface to a temperature sufficient to thermally disrupt or eliminate the biofilm, a much more clinically feasible approach than the immersion methods used in many hyperthermia studies to date. The propensity of the bacteria to disperse might profoundly impact the efficacy of thermal shocks delivered in this manner compared to shocks delivered by directly heating the surroundings. When the surroundings are at least as hot as the biofilm, any dispersing bacteria will be subjected to at least as much thermal shock and may be even more susceptible in the dispersed state. When the surroundings are cooler, however, they could provide a refuge to which the bacteria may temporarily flee, partially evading the shock. Studies on bacterial dispersion from biofilms rarely consider the reverse process, where dispersed bacteria may reattach to the biofilm.

### 4.1. Immersion Thermal Shock

Tobramycin alone did not eliminate *P. aeruginosa* biofilms of either architecture, reducing the population by about two orders of magnitude even at supratherapeutic concentrations, showing no further benefit even with increasing exposure time from 4 and 24 h. Similarly, 5 min 50 °C thermal shocks had no effect on ST biofilms either. When these two approaches were combined, however, the biofilm population density immediately dropped two orders of magnitude and failed to recover over the next 20 h. Thermal shocks at 60 °C for 1 min had a similar result, but with a 2.5 order of magnitude decrease. When the 50 °C thermal shock was extended to 30 min, the immediate population drop was 3.5 orders of magnitude, and some of the replicates then died off entirely over the subsequent 19.5 h. More aggressive thermal shocks (60 °C for 5 min, 70 °C for 1 min), which dropped the population density 2.5 orders of magnitude on their own, and, combined with tobramycin, achieved an immediate 4.5 orders of magnitude decrease. Critically, after a decrease of this magnitude, every replicate died off over the subsequent 20 h. This critical population decrease, from a combination of thermal shock and antibiotics, had previously been demonstrated with ciprofloxacin [26]; these results show that this effect is general to antibiotics in different classes with different modes of action. These effects also appear general to biofilm architecture and initial population density. DFR biofilms with 10 times more initial bacteria drop 2.5 or 3 orders of magnitude when shocked at 70 °C for 1 min or 60 °C for 5 min, respectively, just like ST biofilms. But with these DFR biofilms, adding 4 μg/mL antibiotic exposure has no additional impact on the population density. More aggressive shocks, however (70 °C for 2 min, 60 °C for 10 min), prompting a 3.5 log decrease on their own, also see an additional 1.5 log decrease with tobramycin, followed by elimination of the biofilm over the next 20 h. This indicates a more complicated relationship between mechanisms that would be suggested by simple synergy (i.e., the decrease from the combined approaches being more than the sum of the decreases from each individual approach). Other organisms like *Staphylococcus epidermidis* have shown true synergy between thermal shock and antibiotics [21], indicating that the interaction depends on the organism. Figure 3 and Figure 4 also show that this interaction depends upon more than simply signaling the presence of an antibiotic; subclinical concentration (0.25 μg/mL) had no effect whatsoever, either by itself or in conjunction with thermal shock.

### 4.2. Remote Heating in a Flow Cell vs. Immersion Heating

Immersion is the ideal heating mode for basic thermal susceptibility studies, as it provides precise, uniform heating to the entire biofilm almost instantaneously. It clearly cannot be implemented clinically, however, as delivering the heat through the surrounding tissue would kill all of that tissue, which will typically succumb at a mere 43 °C with prolonged exposure [38,39]. Rather, the goal is to generate the heat directly at the biofilm surface, keeping the biofilm hotter than the surrounding tissue into which the heat will dissipate and ideally be carried away by the circulatory system. Several means of generating heat at the biofilm surface have been proposed, including inductive heating using an alternating magnetic field, allowing external control of the heating without any wires or internal power supply. Any surface heating strategy, however, faces the same potential weakness. If the biofilm’s surroundings are cooler than its surface, those surroundings provide a potential haven in which the bacteria can temporarily reside, evading the thermal shock. Dispersed bacteria are more susceptible to removal by the immune and circulatory systems, however, which may partially mitigate this phenomenon.

This hypothesis was tested by heating the biofilm substrate in a flow cell in which the surrounding media was constantly replaced by media at ambient temperature. Over short exposure times, particularly 1 min, these temperature differences are large, as the temperature profile of the media has not yet been fully developed, as seen in Figure 5. The flowing medium also creates a sink for bacterial removal, as many bacteria are flushed out of the system before they can reattach to the biofilm. This is confirmed in Figure 6, which shows that even when the thermal shock is far too mild to impact the biofilm (e.g., 37 °C control or 60 °C for 1 min), 90% of the bacteria still disappear, a removal rate likely far more aggressive than actually achieved by the immune and circulatory systems. These mild conditions, therefore, serve as controls that isolate the mechanical removal of biofilm by the flowing medium. Nonetheless, this situation still reverses for thermal shocks strong enough to impact the bacteria. At 60 °C for 5 min or 70 °C for 1 min, it appears as though more bacteria are surviving on the biofilm in the flow cell, even though the residence time of the media in the cell is only 20 s before the dispersed bacteria are flushed away. At 70 °C for 5 min or 80 °C for 1 min, this difference becomes significant; biofilms shocked in the flow cell are more populous than those receiving the same shock by immersion. Even with an aggressive convective bacterial sink, these surface-applied thermal shocks leave biofilms with significantly larger population densities than observed in classic immersion trials. This clearly has important implications for treatment, as it directly impacts the severity of the thermal shock that must be applied.

### 4.3. Limitations and Future Directions

Several limitations should be acknowledged. First, the actual rate of removal of dispersed bacteria by the immune system is unknown and possibly quite variable, as is the rate of deactivation by antibiotics. For this study, the flow rate was dictated by balancing the requirements of maintaining a substantial temperature difference while keeping the shear rate low enough to minimize erosive removal of the biofilm, resulting in what may exceed a worst-case-scenario convective removal rate, though that makes any observed increase in post-treatment biofilm population that much more compelling. Second, only one bacterial strain was used for this first study. Different bacteria, particularly without flagella, may have significantly different dispersal and reattachment rates. Similarly, only one antibiotic was investigated to look at its interaction with thermal shock, and it was not incorporated into the flow cell studies, as elimination of dispersed bacteria was already viewed as overly aggressive. Third, this study did not directly assess thermal damage to surrounding tissues. The experimental design focused on the response of biofilms to thermal shock under in vitro conditions and therefore cannot establish the safety profile of the treatment in vivo. Because thermal injury to host tissues is a critical consideration for clinical translation, future studies should quantify temperature distributions within surrounding tissues and evaluate potential thermal damage under clinically relevant conditions. Moving forward, more precise characterization of bacterial dispersion and readhesion rates, as well as the impact of the temperature on these rates, is needed to better characterize the impact of this phenomenon on the efficacy of thermal shock treatment. In addition, evaluation of thermal effects on surrounding tissues will be necessary to assess the clinical feasibility of implant-surface heating strategies.

## 5. Conclusions

Biofilm infections on medical implants remain a major clinical problem that antibiotics cannot resolve by themselves, currently requiring invasive surgical removal and replacement of the device. This study evaluated remote induction heating as a noninvasive approach to eliminate *Pseudomonas aeruginosa* biofilm from a metal surface, together with the ability of tobramycin to reduce the thermal shock required. Tobramycin alone could not eliminate the biofilm of either the shaker table or drip flow reactor architecture, even at very high concentrations. Combined with thermal shock, however, tobramycin achieved complete elimination at milder temperatures and shorter times than thermal shock alone. This aligned well with the hypothesis that if the combined treatment drives down the biofilm population density by a certain critical amount, the remaining biofilm subsequently dies off rather than regrows, as shown previously with antibiotics of a different class and mechanism. Most significantly, this study tested remote induction heating to deliver thermal shocks directly to the biofilm substrate while the surrounding medium remained substantially cooler, as would be the case for in vivo implementation. Despite significant removal of the dispersing bacteria by the flowing media, biofilms shocked directly by their substrate had higher surviving populations than biofilms receiving a comparable shock by immersion in hot surrounding media. While biofilms were completely eliminated by some shocks delivered either way, this study showed that bacterial dispersion and reattachment must be considered when determining the minimum thermal shock to be delivered feasibly by surface heating. Immersion studies likely underestimate the shock required in a clinically feasible localized thermal treatment. These results support remote induction heating as a promising, noninvasive treatment for implant biofilm infections.

## Data Availability

The data supporting the findings of this study are publicly available in the Harvard Dataverse repository at https://doi.org/10.7910/DVN/I4P7AH.

## Abbreviations

The following abbreviations are used in this manuscript:

DFR	Drip flow reactor
ST	Shaker table
TSB	Tryptic soy broth
CFU	Colony-forming unit
NG	No detected growth

## Appendix A

### Appendix A.1

**Table A1:** Combined tobramycin and thermal shock against ST *Pseudomonas aeruginosa* biofilm. Cells show population (log CFU/cm^2^ ± standard deviation). NG = no detectable growth (eliminated). * Growth detected in only 17 of 24 samples.

		Tobramycin				
		No Tobramycin	+0.25 µg/mL (4 h)	+0.25 µg/mL (24 h)	+4 µg/mL (4 h)	+4 µg/mL (24 h)
Thermal shock	37 °C (no shock)	7.13 ± 0.59	7.02 ± 0.17	6.98 ± 0.20	5.64 ± 0.69	6.05 ± 0.65
	50 °C (5 min)	7.27 ± 0.29	6.97 ± 0.34	7.20 ± 0.39	5.47 ± 0.80	5.53 ± 0.46
	50 °C (30 min)	7.28 ± 0.44	6.59 ± 0.18	7.40 ± 0.34	3.40 ± 0.45	3.79 ± 0.97 *
	60 °C (1 min)	7.16 ± 0.62	6.94 ± 0.68	7.14 ± 0.26	4.88 ± 0.79	4.86 ± 0.60
	60 °C (5 min)	4.76 ± 0.79	3.97 ± 0.51	7.10 ± 0.41	2.74 ± 0.30	NG
	70 °C (1 min)	4.91 ± 0.61	5.00 ± 0.63	7.08 ± 0.37	2.94 ± 0.45	NG

### Appendix A.2

**Table A2:** Combined tobramycin and thermal shock against DFR *Pseudomonas aeruginosa* biofilm. Cells show population (log CFU/cm^2^ ± standard deviation). NG = no detectable growth (eliminated).

		Tobramycin				
		No Tobramycin	+0.25 µg/mL (4 h)	+0.25 µg/mL (24 h)	+4 µg/mL (4 h)	+4 µg/mL (24 h)
Thermal shock	37 °C (no shock)	8.46 ± 0.22	8.21 ± 0.31	8.29 ± 0.36	7.10 ± 0.45	6.89 ± 0.49
	60 °C (5 min)	5.48 ± 0.28	5.63 ± 0.44	8.23 ± 0.36	5.54 ± 0.62	5.70 ± 0.73
	60 °C (10 min)	5.07 ± 0.31	5.11 ± 0.38	8.14 ± 0.47	3.83 ± 0.33	NG
	70 °C (1 min)	5.91 ± 0.66	5.82 ± 0.26	8.25 ± 0.18	5.68 ± 0.70	5.31 ± 0.48
	70 °C (2 min)	5.14 ± 0.33	4.95 ± 0.35	7.97 ± 0.39	3.71 ± 0.26	NG
